# Strategien zum rationalen Antibiotikaeinsatz im ambulanten Sektor – Ergebnisse eines Workshops mit wichtigen Akteuren des Gesundheitswesens

**DOI:** 10.1007/s00103-022-03540-z

**Published:** 2022-05-11

**Authors:** Denise Rabold, Muna Abu Sin, Reinhard Bornemann, Alexandra Clarici, Tim Eckmanns, Johannes Hartmann, Julia Hermes, Susanne B. Schink, Roland Tillmann, Ines Perea

**Affiliations:** 1 Referat 615 „One Health, Antimikrobielle Resistenzen“, Bundesministerium für Gesundheit, Unter den Linden 21, 11055 Berlin, Deutschland; 2 FG 37 „Infektionen, Surveillance von Antibiotikaresistenz und -verbrauch“, Robert Koch-Institut, Berlin, Deutschland; 3 Fakultät für Gesundheitswissenschaften, AG2 „Bevölkerungsmedizin und Versorgungsforschung“, Universität Bielefeld, Bielefeld, Deutschland; 4 Ärztenetz Bielefeld, Hausarztpraxis Hartmann & Thomzik, Bielefeld, Deutschland; 5 ABS-Netzwerk Bielefeld – Ostwestfalen-Lippe, Kinder- und Jugendarztpraxis Tillmann, Bielefeld, Deutschland

**Keywords:** Antibiotic Stewardship, Antibiotikaresistenz, Gesundheitspolitik, Interventionsmaßnahmen, Deutschland, Antibiotic stewardship, Antibiotic resistance, Health policy, Intervention measures, Germany

## Abstract

Im November 2021 veranstaltete das Bundesministerium für Gesundheit (BMG) den eintägigen virtuellen Workshop „Rationaler Antibiotikaeinsatz im ambulanten Sektor – Potenziale und Möglichkeiten für Veränderungen“ unter wissenschaftlicher Begleitung des Robert Koch-Instituts (RKI). Ziel war es, geeignete Strategien zur Förderung des sachgerechten Antibiotikaeinsatzes im ambulanten Bereich zusammenzutragen. Mit den 114 Teilnehmenden waren wichtige Akteure des Gesundheitswesens vertreten. Bereits im Vorfeld der Veranstaltung waren die Eingeladenen gebeten worden, an einer Onlinebefragung zu Perspektiven, Erfahrungen und Ideen für den rationalen Einsatz von Antibiotika im ambulanten Sektor teilzunehmen. Die Antworten wurden für den Workshop ausgewertet.

Der Workshop wurde mit Plenarvorträgen zur Deutschen Antibiotikaresistenzstrategie (DART) und zur Antibiotikaresistenzsituation in Deutschland eingeleitet. Alle teilnehmenden Expertinnen und Experten diskutierten in 10 Arbeitsgruppen; deren Ergebnisse wurden in der abschließenden Plenarsitzung vorgestellt. In dem vorliegenden Bericht werden ausgewählte Aspekte dieser Diskussionen präsentiert. Die gewonnenen Erkenntnisse sollen in die Strategie „DART 2030“ einfließen.

## Abstract (en)

In November 2021, the Federal Ministry of Health (BMG) organized the one-day virtual workshop “Rational antibiotic use in the outpatient sector – potential and opportunities for change” with scientific support from the Robert Koch Institute (RKI). The aim was to collect strategies for promoting the appropriate use of antibiotics in the outpatient sector. With 114 participants, important stakeholders of the healthcare system were represented. In the run-up to the event, the invited participants had already been asked to take part in an online survey on perspectives, experiences, and ideas for the rational use of antibiotics in the outpatient sector. The answers were analyzed and presented at the workshop.

The workshop was introduced with plenary lectures on the German Antibiotic Resistance Strategy (DART) and the antibiotic resistance situation in Germany. All experts participated in 10 working group discussions; the resulting findings were presented in the concluding plenary session. In this conference report, selected aspects of these discussions are presented. The insights gained are to be incorporated into the “DART 2030” strategy.

Im Rahmen der jährlich durch die Weltgesundheitsorganisation (WHO) initiierten „World Antimicrobial Awareness Week“ fand am 18.11.2021 ein vom Bundesministerium für Gesundheit (BMG) organisierter ganztägiger virtueller Workshop „Rationaler Antibiotikaeinsatz im ambulanten Sektor – Potenziale und Möglichkeiten für Veränderungen“ statt. Ziel des Workshops war es, Aktivitäten und Strategien für einen rationalen Antibiotikaeinsatz (Antibiotic Stewardship – ABS) im ambulanten Bereich zu identifizieren. Zudem sollten Ergebnisse aus kürzlich abgeschlossenen und noch laufenden Projekten reflektiert werden. Chancen und Potenziale, aber auch Herausforderungen für die Implementierung dieser Erkenntnisse sollten mit Beteiligten aus Wissenschaft und Praxis sowie Entscheidungstragenden aus dem Gesundheitswesen diskutiert werden.

Das Interesse am Workshop war sehr hoch und reflektierte die Relevanz der Thematik sowie die Bereitschaft für Veränderungen hin zu einem bestmöglichen sachgerechten Antibiotikaeinsatz. Die 114 Teilnehmenden repräsentierten ein breites Spektrum von Akteuren des deutschen Gesundheitswesens: Vertreterinnen und Vertreter des ambulanten Sektors, der kassenärztlichen Vereinigungen (KVen), der Kassenärztlichen Bundesvereinigung (KBV), des Zentralinstituts für die kassenärztliche Versorgung (Zi), der gesetzlichen Krankenkassen (KKen), des GKV-Spitzenverbands, der medizinischen Fachgesellschaften, der Arbeitsgemeinschaft der Wissenschaftlichen Medizinischen Fachgesellschaften (AWMF), der MRE^1^-Netzwerke, der Bundesärzte- bzw. -zahnärztekammer, der Apothekerverbände, der Bundeszentrale für gesundheitliche Aufklärung (BZgA), Vertreterinnen und Vertreter aus den Ländern, des Aktionsbündnisses Patientensicherheit e. V., des Instituts für Qualitätssicherung und Transparenz im Gesundheitswesen (IQTIG), des lokalen und regionalen Öffentlichen Gesundheitsdienstes, des Bundesinstituts für Arzneimittel und Medizinprodukte (BfArM), des Robert Koch-Instituts (RKI), des Bundesministeriums für Bildung und Forschung (BMBF) und des BMG.

## Workshopauftakt und Plenarvorträge

In seinem Grußwort betonte Dr. Ulrich Holtherm (BMG), ehemaliger Leiter^2^ der Abt. 6 „Gesundheitsschutz, Gesundheitssicherheit, Nachhaltigkeit“, die Relevanz der gemeinsamen Bestrebungen eines sachgerechten Antibiotikaeinsatzes. Thematisch führte anschließend Ines Perea (BMG), Leiterin des Ref. 615 „One Health, Antimikrobielle Resistenzen“, in die Veranstaltung ein und informierte über die Fortschreibung der Deutschen Antibiotika-Resistenzstrategie [1] als „DART 2030“. Zur aktuellen Resistenzsituation basierend auf Daten der Antibiotika-Resistenz-Surveillance (ARS) am RKI berichtete Dr. Tim Eckmanns (RKI), Leiter des Fachgebiets 37 „Nosokomiale Infektionen, Surveillance von Antibiotikaresistenz und -verbrauch“, dass Deutschland einerseits bei den Resistenzanteilen von Erregern wie *S. aureus *(MRSA) oder *E. coli* mit Resistenz gegen Cephalosporine der 3. Generation in den letzten Jahren absteigende Tendenzen zu verzeichnen habe. Andererseits warnte er jedoch vor einem ansteigenden Trend bei Vancomycin-resistenten Enterokokken (VRE), welche insbesondere im stationären Bereich Sorge bereiten.

## Ergebnisse aus Forschungsprojekten

Im Anschluss an die Plenarvorträge wurde der Fokus auf 8 Forschungsprojekte gelenkt, welche sich zunächst jeweils per Kurzfilm vorstellten:

- Antibiotika-Resistenzentwicklung nachhaltig abwenden: ARena^3^,
- Antibiotische Therapie in Bielefeld: AnTiB^4^,
- Rationaler Antibiotikaeinsatz durch Information und Kommunikation: RAI^5^,
- Reduktion von Antibiotikaresistenzen: RedAres^6^,
- Resistenzvermeidung durch adäquaten Antibiotikaeinsatz bei akuten Atemwegsinfektionen: RESIST^7^,
- Surveillance des ambulanten Antibiotikaeinsatzes: SAMBA^8^,
- Studie zur Analyse der regionalen Unterschiede bei der Antibiotika-Verordnung: SARA^9^,
- Verbesserung des Umgangs mit Antibiotika bei akuten Atemwegsinfekten in der deutschen Primärversorgung: CHANGE‑3^10^ [2].

In der folgenden Projektübersicht wurden die Erkenntnisse dieser Studien herausgearbeitet, wie etwa unterschiedliche Interventionsmaßnahmen, die zu einer Reduktion im Antibiotikaverbrauch beitragen können, beispielsweise: die Entwicklung von Praxismaterialien für Patientinnen und Patienten und Schulungsmaterialien für Ärztinnen und Ärzte; Feedbacksysteme für Ärztinnen und Ärzte zum eigenen Verordnungsverhalten und zum Vergleich mit anderen; Förderung der Zusammenarbeit in Peergruppen, Netzwerken und Qualitätszirkeln; potenzielle Vergütungsanreize; die Entwicklung von Leitlinien und die Optimierung bestehender Handlungsempfehlungen sowie die Optimierung des Verordnungsverhaltens im regionalen und sozialen Kontext.

Als Praxismaterialien für Patientinnen und Patienten haben sich mehrsprachige Printmedien bewährt, da damit Zielgruppen, wie zum Beispiel Menschen mit Migrationshintergrund oder auch jüngere Bevölkerungsgruppen, besser erreicht werden können. Diskutiert wurde eine verstärkte Einbindung von sozialen Medien und speziell von „Influencern“. Bei praktisch tätigen Ärztinnen und Ärzten haben sich zertifizierte Onlinefortbildungen als besonders beliebt herausgestellt. Zudem wurde deutlich, dass ihr Mitwirken auf lokaler Ebene in ABS- bzw. MRE-Netzwerken oder Qualitätszirkeln, aber auch regelmäßige Feedbackberichte zum eigenen Verschreibungsverhalten im Vergleich mit anderen Ärztinnen und Ärzten wichtige Anreize darstellen. Hervorgehoben wurde auch die Problematik des Zusammenspiels aus Erwartungshaltung und Antibiotikaverordnung in der Arzt-Patienten-Kommunikation. Während eine entsprechende ärztliche Schulung häufig zunächst mit einer schlechten Aufwand-Nutzen-Bilanz bewertet wird, wären im Nachgang oft Erfolge zu verzeichnen.

## Ergebnisse der Vorab-Onlinebefragung

Vor dem Workshop waren die eingeladenen Expertinnen und Experten gebeten worden, an einer Onlinebefragung zu Perspektiven, Erfahrungen und Ideen für den rationalen Einsatz von Antibiotika im ambulanten Sektor teilzunehmen. Die 127 eingegangen Antworten zeigten die große Bandbreite von Ansätzen, die zu einem sachgerechteren Antibiotikaeinsatz führen könnten. Großen Konsens fand der Ansatz, das Verschreiben von Reserveantibiotika an das Vorliegen eines Antibiogramms^11^ zu knüpfen. Eine Unterstützung durch digitale Anwendungen, z. B. in Form von Apps, und die Einbindung von künstlicher Intelligenz im Praxisalltag wurden als zukunftsweisend erachtet. Um Verordnungsanalysen zum Verschreibungsverhalten von Ärztinnen und Ärzten für einen sachgerechteren Antibiotikaeinsatz anzuwenden, sahen die Mitwirkenden die KKen und die KVen in der Pflicht, Daten z. B. in der Form von individuellen Feedbackberichten bereitzustellen.

Eine bundesweite übergeordnete Surveillance von Antibiotikaverbrauch und Antibiotikaresistenz sollte hierfür Referenzdaten liefern und Trends im zeitlichen Verlauf abbilden, um Entscheidungstragende zu informieren. Darüber hinaus sind Daten zur Resistenzsituation eine wichtige Grundlage für Fachgesellschaften, um Leitlinien und Empfehlungen zeitnah anpassen zu können.

Der Wunsch nach Synopsen von Leitlinien und Empfehlungen wurde bei der Onlinebefragung deutlich; dafür wurden die Fachgesellschaften in der Verantwortung gesehen. Weiterhin wurde empfohlen, das Thema ABS in der medizinischen Aus‑, Weiter- und Fortbildung sowie in angrenzenden Disziplinen, wie etwa der Pflege, weiter praxisnah zu verankern. In der Allgemeinbevölkerung sollte grundlegendes Gesundheitswissen zu Antibiotika zielgruppengerecht gestärkt werden. Die KKen und KVen wurden als wichtige Akteure gesehen, um den Einsatz und die angemessene Vergütung von Schnelldiagnostik (Point-of-Care-Testung) zu ermöglichen, Resistenztestungen und Beratungsgespräche zu implementieren sowie Projekte zum rationalen Antibiotikaeinsatz weiter zu fördern und erfolgreiche Interventionen regional zu verstetigen. Die „verzögerte Verschreibung“, bei der Rezepte erst unter bestimmten Voraussetzungen, z. B. einer Verschlechterung der Symptome, eingelöst werden sollen, sowie die Einrichtung einer „24/7-ABS-Hotline“ wurden als potenzielle Maßnahmen zur nachhaltigen Senkung des Antibiotikaeinsatzes und zur Optimierung der Verordnungspraxis eingestuft.

## Arbeitsgruppen

Während des Workshops kamen Expertinnen und Experten mit verschiedenen fachlichen Hintergründen in Arbeitsgruppen zusammen, die sich mit 10 Schwerpunkten befassten:

1. regionale Netzwerke,
2. Maßnahmen/Anreize zum rationalen Antibiotikaeinsatz durch die Ärzteschaft,
3. Monitoring/Surveillance des Antibiotikaverbrauchs,
4. Öffentlichkeitsarbeit – Wie kann die Kommunikation erleichtert werden?
5. Leitlinien und Praxisalltag,
6. Digitalisierung und künstliche Intelligenz als Unterstützung einer rationalen Antibiotikatherapie,
7. Therapie: Diagnostik verbessern?
8. Verstetigung von Projektergebnissen,
9. mögliche Maßnahmen, die das persönliche Verschreibungsverhalten beeinflussen können,
10. Aus‑, Weiter- und Fortbildung.

Die Diskussion in den Arbeitsgruppen wurde anhand von Leitfragen geführt und ermöglichte einen tiefergehenden Austausch zu den Ergebnissen der vorgestellten Forschungsprojekte und der Vorab-Umfrage.

Die wichtigen Erkenntnisse und Impulse aus dem gesamten Workshop sind in Infobox 1 zusammengefasst.

## Fazit

Unter dem vielfältigen Erkenntnisgewinn der Teilnehmenden ist hervorzuheben, dass insbesondere solche Interventionen einen Fortschritt hin zu einem sachgerechten Antibiotikaeinsatz unterstützt haben, die an lokale Strukturen und Besonderheiten sowie an spezifische Akteurs- und Adressatenkreise angepasst wurden. Erfolgreiche Beispiele dafür fanden sich unter den vorgestellten Projekten und Forschungsvorhaben. Für den wichtigen Austausch und die Interaktion der unterschiedlichen Akteure des Gesundheitswesens können Veranstaltungen wie dieser Workshop einen wertvollen Beitrag leisten. Die Verstetigung und Finanzierung erfolgreicher Projekte sowie deren Ausweitung bleibt aufgrund der komplexen Förderstruktur im deutschen Gesundheitswesen weiterhin eine Herausforderung. Die Mehrheit der Teilnehmenden wünschte sich eine Fortführung der Veranstaltung im zweijährlichen Rhythmus.

### Infobox 1 Wichtige Erkenntnisse und Impulse aus der Tagung

- Verschiedene Informationsformate in einem Methodenmix (analog und digital) können bedarfsgerecht einen wertvollen Beitrag zur Wissensvermittlung an Ärztinnen und Ärzte sowie Patientinnen und Patienten leisten.
- Die Kooperation von Ärztinnen und Ärzten in lokalen Qualitätszirkeln und ABS- bzw. MRE-Netzwerken, auch fachgruppen- und sektorübergreifend, unterstützt die Implementierung von aktuellen Kenntnissen zur rationalen antibiotischen Therapie.
- Eine bundesweite (integrierte) Surveillance von Antibiotikaverbrauch und -resistenz ist notwendig, um Verordnungsverhalten einordnen und Resistenzentwicklungen erkennen zu können.
- Regelmäßige Feedbackberichte mit Vergleichswerten (Benchmarking) im ambulanten ärztlichen Bereich, regional und fachgruppenspezifisch sowie idealerweise mit Daten zur lokalen Resistenzsituation aufgearbeitet, ermöglichen eine Reflexion des eigenen Verordnungsverhaltens.
- Eine verbesserte Arzt-Patienten-Kommunikation kann unsachgemäßem Antibiotikaverbrauch entgegenwirken und das Vertrauensverhältnis stärken.
- CME-zertifizierte Onlineschulungen sind beliebt; eine regelmäßige inhaltliche und technische Aktualisierung sowie finanzielle und personelle Ausstattung sollten gewährleistet sein.
- Kurzgefasste, praxistaugliche bzw. anwendungsfreundliche Empfehlungen zum indikationsgerechten Antibiotikaeinsatz auf Basis existierender Leitlinien sowie ggf. ergänzende digitale Unterstützungstools sollten ausgebaut werden.
- Die Beratung und die sachgerechte Verordnung benötigen einen erhöhten Aufwand, welcher angemessen honoriert werden sollte.
- Ärzte mit einer hohen Rate an Viel- und Fehlverordnungen sind bisher durch verschiedene Schulungsangebote und Kampagnen schwer zu erreichen gewesen; KKen und KVen können hier mit Angeboten unterstützen.

## Footnotes

1 MRE: multiresistente Erreger.

2 Bis zum Dezember 2021.

3 Gefördert 01/2017 bis 09/2020 durch den Innovationsfond, Gemeinsamer Bundesausschuss; https://antibiotika-alternativen.de/ (20.04.2022); zu Projektdetails vgl. [2].

4 Gefördert durch Eigenmittel; www.antib.de (20.04.2022); zu Projektdetails vgl. [2].

5 Gefördert 02/2015 bis 06/2022 durch das Bundesministerium für Bildung und Forschung; www.rai-projekt.de (20.04.2022); zu Projektdetails vgl. [2].

6 Gefördert 09/2019 bis 03/2022 durch den Innovationsfond, Gemeinsamer Bundesausschuss; www.redares.de (20.04.2022).

7 Gefördert von 12/2016 bis 04/2020 durch den Innovationsfond, Gemeinsamer Bundesausschuss; https://www.kbv.de/html/resist.php (20.04.2022); zu Projektdetails vgl. [2].

8 Gefördert von 04/2019 bis 05/2022 durch das Bundesministerium für Gesundheit; https://www.rki.de/DE/Content/Infekt/Antibiotikaresistenz/RKI-Aktivitaeten/SAMBA.html (20.04.2022).

9 Gefördert von 04/2019 bis 03/2022 durch das Bundesministerium für Gesundheit; https://www.bips-institut.de/forschung/projekte/einzelansicht.html?projID=786 (20.04.2022).

10 Gefördert von 02/2017 bis 06/2020 durch das Bundesministerium für Gesundheit; www.weniger-antibiotika.de (20.04.2022); zu Projektdetails vgl. [2].

11 Antibiogramm: Darstellung der Empfindlichkeit eines isolierten Bakteriums gegenüber antibiotischen Wirkstoffen; Resistenz‑/Sensitivitätstestung.
